# Novel *GRN* Mutations in Patients with Corticobasal Syndrome

**DOI:** 10.1038/srep22913

**Published:** 2016-03-10

**Authors:** Foad Taghdiri, Christine Sato, Mahdi Ghani, Danielle Moreno, Ekaterina Rogaeva, Maria Carmela Tartaglia

**Affiliations:** 1 Tanz Centre for Research in Neurodegenerative Diseases, University of Toronto, 60 Leonard avenue, Toronto, ON M5T 2S8, Canada; 2 Department of Medicine, Division of Neurology, University of Toronto, 1 King’s College Circle, Toronto, ON M5S 1A8, Canada; 3 Devision of Neurology, University Health Network memory clinic, Toronto Western Hospital, 399 Bathurst street, ON M5T 2S8, Canada

## Abstract

Loss-of-function *GRN* mutations lead to GRN haploinsufficiency and consequently neurodegeneration with significant heterogeneity in clinical presentation of various syndromes. The aim of this study was to investigate the genetics and clinical features of patients with *GRN*-related frontotemporal lobar degeneration (FTLD) syndromes. We performed mutation analysis of *GRN* in 45 unrelated Canadian patients with a broad spectrum of FTLD-like syndromes (mean age at onset of 64.0 ± 11.2 years). In our cohort, two patients were carriers of two novel heterozygous alterations in *GRN*: 2 bp insertion (c.769–770insCC:p.Q257fs) and 12 bp deletion (c.1009–1020del:p.337–340del). Both patients presented with corticobasal syndrome supported by clinical and radiological findings. The absence of the mutant allele in the RT–PCR product was only observed for the sample with 2 bp insertion in *GRN*. In contrast, the allele with 12 bp deletion in *GRN* was not down-regulated at the RNA level and did not segregate with FTLD in the family. Our report extends the evidence for genetic and phenotypic variability in FTLD disorders, and detects a novel pathogenic *GRN* mutation, carriers of which could eventually help to evaluate the efficacy of different treatments at early stages of dementia.

Progranulin (GRN), encoded by the *GRN* gene on chromosome 17q21, is a multifunctional growth factor, which is expressed in the brain by microglia and neurons, and is critical in maintaining neuronal survival^1^. Loss-of-function *GRN* mutations lead to GRN haploinsufficiency and consequently neurodegeneration^2^. These mutations are associated with high phenotypic variability and have been identified in a number of neurodegenerative syndromes that fall under the umbrella term of frontotemporal lobar degeneration (FTLD), including behavioral variant frontotemporal dementia (bvFTD), primary progressive aphasia (PPA), and corticobasal syndrome (CBS)^3^,^4^. Mutations in *GRN* comprise a significant cause of FTLD, accounting for 5–11% of sporadic patients and more than 20% of familial FTLD worldwide^5^,^6^. To date, 71 pathogenic *GRN* mutations have been identified, which are expected to cause haploinsufficiency by nonsense-mediated decay (http://www.molgen.vib-ua.be/FTDMutations). In addition, there are 80 reported variants that are missense substitutions, which are either not pathogenic (n = 46) or their pathogenicity is unclear (n = 34).

The clinical, neuropsychological, and neuroimaging findings of FTLD associated with *GRN* mutations are variable. Behavioral changes, especially apathy and social withdrawal, are the most common symptoms^7^. Less spontaneous speech, word finding difficulties, mutism, and phonemic paraphasias are also prominent clinical features of carriers of *GRN* mutations^8^. In addition, cognitive dysfunction and progressive non-fluent aphasia tend to be more frequent in patients with CBS caused by *GRN* mutations.

CBS is a syndrome that usually begins in the sixth decade of life and its clinical manifestations can be explained by the involvement of cortical and subcortical structures. Patients with CBS typically present with progressive asymmetric rigidity, apraxia, extrapyramidal dysfunction such as alien-limb phenomenon, cortical sensory loss, bradykinesia, myoclonus, and focal dystonia^9^.

Herein, we describe the clinical, radiological, and genetic findings in a cohort of Canadian FTLD patients, including two with novel *GRN* mutations, which extend the knowledge on the phenotypic variability and mutation spectrum of *GRN*.

## Results

### Genetic findings

**Figure 1:**
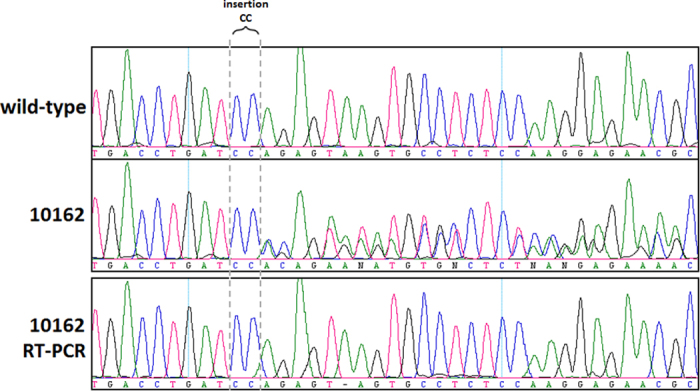
Mutation analysis of *GRN* in individual 10162 diagnosed with CBS. The sequence chromatogram shows the insertion of CC in 10162 below a wild-type sequence. The bottom panel shows the RT-PCR sequence results for 10162; the deletion was not detected.

Two of the 45 investigated FTLD samples (ID 10162 and ID 9957) revealed heterozygous genetic alterations in *GRN*. Mutations in the other two most common FTLD genes (*MAPT* and *C9orf72*) were excluded in our subjects^10^,^11^. In patient 10162 we detected a novel frame-shift 2 bp insertion (CC) in exon 8 (*GRN*:NM_002087:exon8:c.769–770insCC:p.Q257fs). It is predicted to truncate the protein distal to the mutation site with a stop codon at amino acid position 283, likely leading to haploinsufficiency of the GRN protein. Indeed, we confirmed the absence of the mutant allele in the RT–PCR product of sample 10162 (Fig. 1).

**Figure 2:**
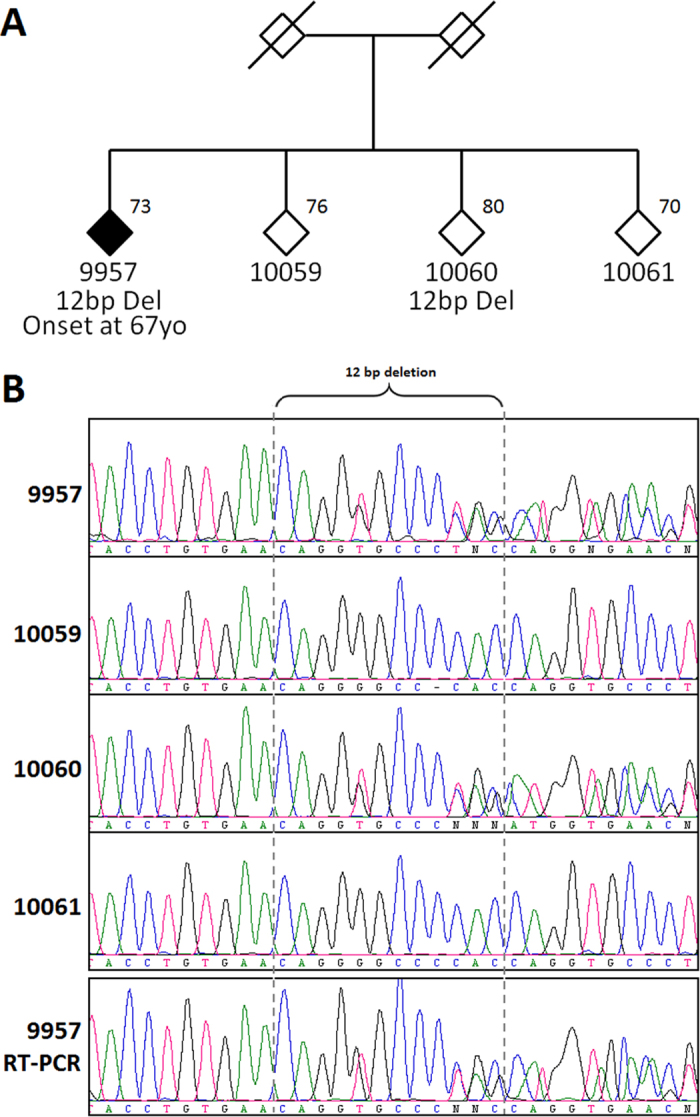
Mutation analysis of *GRN* in individual 9957. (**A**) The family diagram of 9957 diagnosed with CBS (filled symbol). Age at time of blood draw is specified at the top right where available. Gender is masked to protect family confidentiality (a slash indicates deceased persons). (**B**) The sequence chromatogram shows the 12 bp deletion observed in 9957 and sibling 10060, but not in siblings 10059 and 10061. The bottom panel shows the RT-PCR sequence results for 9957; the deletion was detected.

In patient 9957 we detected a novel in frame 12 bp deletion (CAGGGGCCCCAC) in exon 10 (*GRN*:NM_002087:exon10:c.1009_1020del:p.337–340del). Although this deletion does not affect a conserved region, it causes the loss of four amino acids (QGPH at p.337–340) in the Granulin-4 section of the protein (FTId = PRO_0000012698) and might disrupt the secondary and tertiary structure of GRN (according to MutationTaster), potentially affecting its interaction with Elastase^12^. However, our results do not support the pathological nature of this variant. To validate if the deletion affected a cryptic splicing site leading to haploinsufficiency we conducted RT-PCR, which revealed that the mutant allele is expressing at similar levels to the wild-type allele. Furthermore, we found the same *GRN* mutation in one of the siblings of patient 9957, who is now 82 years old and has no signs of FTLD (Fig. 2).

### Clinical findings

#### CBS patient 10162 with a 2 bp insertion in *GRN*

This 68-year-old, right-handed woman presented at age 67 with an insidious onset of cognitive changes that had started 2 years prior. The first sign was some hesitancy in leaving the house by herself because of a fear of getting lost. Her memory worsened and she began to leave doors unlocked and lights on. There had been a more rapid progression in the last six months prior to the first assessment, when she had begun to have difficulty with basic activities of daily living such as dressing (e.g. could no longer put on her bra or recognize the right from the left shoe). The patient had difficulty recalling recent events, had become more repetitive and often misplaced items in the home. There were notable language problems such as word-finding difficulty, comprehension deficits and stammering. In the 2 years prior to presentation, she had difficulty navigating both in familiar and unfamiliar surroundings. The patient stopped driving because she was incapable of making right turns. She had personality changes having become apathetic and irritable. She often needed prompting to perform any task. The patient had difficulty with some instrumental activities of daily living such as finances (e.g. paying $1000 instead of $100) and had difficulty doing housework. There were no gait disturbances or falls; however, she had difficulty going down the stairs. There were no tremors or other abnormal movements. Her neuropsychological testing (Table 1) revealed severe cognitive impairment in multiple domains, worse in visuospatial function but both verbal and visual memory were impaired as well as executive function and language. On physical exam, she exhibited mild parkinsonism, ideational and ideomotor apraxia and neglect on the left.

In the following year, her language comprehension continued to decline. The patient perseverated and became fixated on certain ideas. There was worsening bradykinesia in the left hand and leg. The patient’s past medical history included only hypertension and hyperlipidemia. Her family history included alcoholism in her father but no other neurodegenerative disease. She had no knowledge of her dad’s family history but on her mother’s side, her relatives had lived into their 80 s without any cognitive problems.

**Figure 3:**
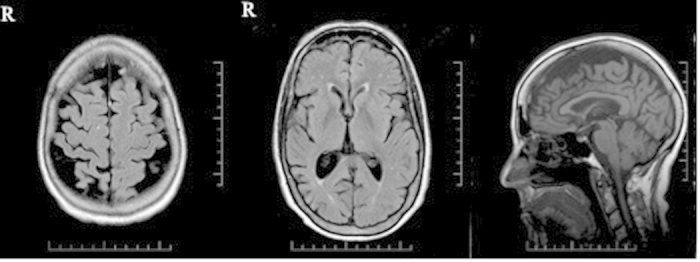
T1-weighted MRI of the brain of patient 10162.

MRI of the brain performed at 67 years of age (2 years after symptom onset) of patient 10162 showed asymmetrical, right greater than left frontoparietal atrophy (Fig. 3).

#### CBS patient 9957 with a 12 bp deletion in *GRN*

This 74-year-old, right-handed man gave a 5-year history of slowness in gait and right limb movement. He had difficulty with stairs, especially going downstairs, and he had fallen a number of times. In addition, he developed changes in personality; severe apathy and loss of empathy for his family. His thinking was slower. In terms of memory, he often repeated himself, and could not recall recent conversations although remote memory was intact. He had difficulty with planning and organizing, as well as with problem solving. His initial language problems included substitution of words in speech, anomia, stuttering, dysarthria, and a dramatic decrease in speech output. The decline in his language function had progressed significantly until he became almost completely mute. His neuropsychological assessment revealed severe language and executive deficits with moderate-severe verbal memory deficits and relative preservation of visuospatial function(Table 1). He had become apraxic for using utensils, but continued to dress himself. There was posturing in the right foot. He was a 15-pack-year smoker that had quit years ago. He used to drink 2 alcoholic beverages per day, but no longer drank alcohol. He reported no recreational drugs. There was no history of a neurodegenerative disease in his family (father died at age 85 of unknown causes; mother died at 63 of congestive heart failure). He had four siblings with no neurodegenerative disease. *GRN* mutation testing was performed in two brothers (76 and 80 years old) and his sister who was 70. The 80-year-old brother who is still free of any clinical evidence of a neurodegenerative disease, continuing to manage independently all Instrumental Activities of Daily Living (IADLs) including driving, has the same *GRN* mutation as the patient. His 48-year-old son and 46-year-old daughter are both healthy.

**Figure 4:**
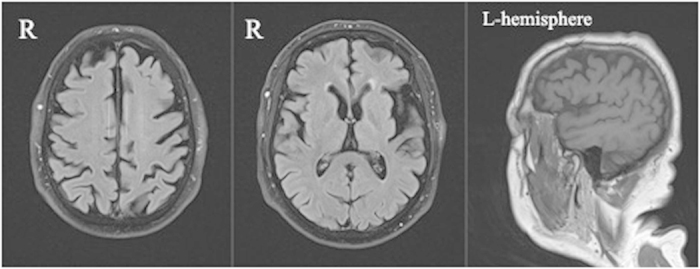
T1-weighted MRI of the brain of patient 9957.

MRI of the brain acquired at age 72 (3 years after symptom onset) revealed asymmetrical, left greater than right, frontoparietal atrophy. The greatest involvement was the left inferior frontal gyrus. (Fig. 4)

## Discussion

### We identified two novel *GRN* mutations in two patients clinically diagnosed with CBS

Clinical symptoms of case 10162 began with a gradual decline in her cognition including comprehension, memory, executive and visuospatial impairment. This progressed to apraxia and neglect on her left side, and parkinsonism. The genetic study demonstrated a 2 bp insertion in exon 8. There is no doubt about the pathological nature of this frameshift mutation, since RT-PCR demonstrated that the mutation results in haploinsufficiency. Although patient 10162 had no positive family history of a neurodegenerative disease, the patient’s father died at a young age (54 due to alcoholism), which might indicate FTLD since addiction has been reported in these syndromes^13^. However, this could be due to a *de novo GRN* mutation or the reduced penetrance of *GRN* mutations that are often associated with the wide range in age of onset^14^,^15^. Even in a single family, age at onset in the carriers of *GRN* mutation could be more than five decades apart^16^.

The second patient with CBS (ID 9957), presented initially with motor symptoms consisting of asymmetric parkinsonism, right-sided dystonia, and falls as well as prominent apathy, short-term memory loss, language impairment and executive dysfunction. In contrast to patient 10162, subject 9957’s disease is unlikely related to a *GRN* mutation because the detected novel 12 bp deletion in *GRN* did not segregate with FTLD in the family of patient 9957; and the mutant allele is expressed at similar levels to the wild-type allele. However, one of the limitations of our study is the absence of blood plasma to estimate the levels of progranulin protein. This is noteworthy because there are reports of rare missense *GRN* mutations (e.g. A266P and C126W) that are associated with a moderate decrease of GRN protein which could contribute to disease risk^17^.

Neuropsychological testing in patients with CBS usually reveals executive and visuospatial deficits, domains related to frontal and parietal cognitive networks but sometimes significant language impairment is also seen as in our patients (Table 1)^9^. Both of our patients showed MRI patterns typical of CBS, which is an asymmetric frontoparietal atrophy that usually involves the supplementary motor area and parietal regions^8^.

The majority of cases of CBS are sporadic, however, familial cases are described in some studies. For instance, the genetic basis of the CBS in some families has been associated with mutations either in microtubule associated protein tau (*MAPT*) gene or in *GRN*^18^. In rare cases, *C9orf72* mutations can present with CBS^19^,^20^,^21^.

Although clinical findings overlap between the *GRN*- and *MAPT*-dependent phenotypes, they are completely different at the cellular level. In the *GRN*-linked condition, there is cellular aggregation and mislocalization of TAR DNA/RNA binding protein 43 (TDP-43). TDP-43 is expressed in many tissues and plays a role in transcription regulation, exon skipping, and some other cellular processes. Mutations in *GRN* cause caspase-dependent cleavage of TDP-43, which consequently leads to the accumulation of TDP-43 fragments^8^. Although tau pathology is the most frequent pathological substrate in CBS, other pathologies including Alzheimer’s disease (amyloid and tau), PSP, TDP-43 and even Creutzfeldt-Jakob disease can cause the disease. The genetics of CBS is largely unknown; *GRN* mutations have also been described in sporadic forms of FTLD including in CBS^9^. About 5–20% of all familial FTLD cases and 1–5% of sporadic cases are caused by mutations in *GRN*^22^,^23^, whereas, *MAPT* mutations are very rare in sporadic forms of FTLD^8^. Le Ber *et al.* published the first study evaluating the frequency of *GRN* mutations in patients with PPA and CBS in 2008, and reported that 3.3% of their CBS patients were *GRN* mutation carriers. They found *GRN* mutations in a proband and her sister who both presented as CBS with behavioral symptoms at onset that rapidly progressed to asymmetric parkinsonism including rigidity and apraxia in the left upper limb^24^. Several other studies have shown an association between *GRN* and CBS^25^,^26^. The study by Almeida *et al.* described a sporadic CBS patient presenting with a frameshift g.2263–2264dupGT mutation^27^. Benussi *et al.* presented two Italian families with variable clinical phenotypes including CBS^28^. In addition, Masellis *et al.* reported a novel splice donor site mutation that segregated with CBS in Canadian family of Chinese origin^29^.

In conclusion, genetic variation in *GRN* is linked to various familial FTLD syndromes with heterogeneity in presentation and syndrome. GRN haploinsufficiency leads to the accumulation of pathological TDP-43 inclusions although the mechanism is largely unknown^8^. A better understanding of the relationship between GRN haploinsufficiency and TDP-43 pathology could lead to potential treatments for FTLD syndromes associated with TDP-43 pathology. This study shows the importance of investigating more extensively any genetic alterations found in patients with FTLD and possibly other neurodegenerative diseases.

## Methods

### Data set

We performed mutation analysis of *GRN, MAPT,* and *C9orf72* in 45 unrelated cases. Our cohort comprised of patients with a broad spectrum of FTLD-like syndromes diagnosed according to the latest criteria^30^,^31^,^32^ with a mean age at onset of 64.0 ± 11.2 years (37.8% female). It included 8 patients with probable bvFTD, 3 patients with frontotemporal dementia with motor neuron disease (FTD-MND), 21 patients with CBS, 11 patients with probable semantic variant PPA, and 3 patients with progressive supranuclear palsy (PSP). Informed consent was obtained from all participants in accordance with the University of Toronto ethics review board and all experimental protocol were approved by this review board.

### Genetic analysis

Genomic DNA and total RNA were extracted from whole blood using Qiagen kits. The entire open reading frame, including exon-intron boundaries of *GRN* was analysed by Sanger sequencing as previously described^33^. RT–PCR primers for patient 10162 were designed for *GRN* exon 7 (5′-AGTGGGAAGTATGGCTGCTG-3′) and exon 9 (5′-TAGACGGCAGCAGGTATAGC-3′); and RT–PCR primers for patient 9957 were designed for *GRN* exon 9 (5′-GCTATACCTGCTGCCGTCTA-3′) and exon 10 (5′-TCTTCAAGGCTTGTGGGTCT-3′). The RT–PCR conditions were 94 °C for 5 min, followed by 40 cycles of 94 °C for 30 s, 58 °C for 30 s, 72 °C for 30 s, and 7 min at 72 °C. Mutations were detected by direct inspection of the fluorescent chromatographs and by analysis using the SeqScape software version 1.0 (Applied Biosystems, FosterCity, CA).

## Additional Information

**How to cite this article**: Taghdiri, F. *et al.* Novel *GRN* Mutations in Patients with Corticobasal Syndrome. *Sci. Rep.* **6**, 22913; doi: 10.1038/srep22913 (2016).

## Figures and Tables

**Table 1:** Demographics and neuropsychological assessment scores of the two CBS patients with GRN mutations.

Demographics, neuropsychological assessment (test name/maximum score)	Case 10162	Case 9957
Age of onset, years	64	69
Age at this testing, years	68	72
Duration of disease at testing, years	4	3
Orientation/12	5/12	7/12
Memory		
CERAD word list trial one/10	2/10	1/10
CERAD word list trial two/10	4/10	2/10
CERAD word list trial three/10	5/10	3/10
CERAD delayed recall/10	3/10	1/10
CERAD delayed recognition/20	12/20	16/20
Benson figure delayed recall/17	0/17	8/17
Benson figure delayed recognition/1	0/1	1/1
Executive functions/concentration/attention		
Serial sevens/13	0/13	1/13
Serial threes/13	0/13	4/13
Digit span—forward/9	6/9	4/9
Digit span—backward/8	2/8	0/8
TMT-A	0	0
TMT-B	0	0
Alternating sequences/2	0/2	1/2
Similarities/10		
Visuospatial function		
Benson figure copy/17	0/17	15/17
Clock drawing/15		
Language function		
Verbal fluency		
F-words	2	0
Animals	6	0
MINT naming/15	10/15	8/15
Repetitions/10	6/10	4/10
Single word comprehension/8	4/8	8/8
Single word reading comprehension/2	1/2	2/2
Sentence comprehension/8	6/8	2/8
Single word reading/12	12/12	4/12
Semantic knowledge/10	4/10	2/10

*Abbreviations:* CERAD = Consortium to Establish a Registry for Alzheimer’s Disease; MINT = Multilingual Naming test; TMT= Trail Making Test.
